# Electric Field Modulation Enables High Energy-Storage Density and Low Loss in All-Organic Polymer Films with Gradient Dielectric Constants

**DOI:** 10.3390/polym18182202

**Published:** 2026-09-10

**Authors:** Jianfeng Li, Zerui Li, Fujia Chen, Yujiu Zhou, Hu Ye, Zhenyi Qu, Yuetao Zhao, Jianhua Xu

**Affiliations:** 1 School of Optoelectronic Science and Engineering, University of Electronic Science and Technology of China, Chengdu 610054, China; ljf1510830@163.com (J.L.); zeruili022@163.com (Z.L.); chem24@163.com (H.Y.); 2 Department of Film Capacitors, Xinyun Electronic Components Company of China ZhenHua Group, Guiyang 550018, China; edc0402@163.com (F.C.); 18782941993@163.com (Y.Z.); 3 School of Creative Design, Chongqing City Vocational College, Chongqing 402160, China; qzyym1217@163.com; 4 Ocean College, Jiangsu University of Science and Technology, Zhenjiang 212100, China

**Keywords:** gradient of the dielectric constant, energy storage, control of interface traps, charge and discharge efficiency

## Abstract

Achieving a balance between high energy-storage density and high charge/discharge efficiency remains a key challenge for polymer film capacitors. In this study, a gradient-dielectric all-organic sandwich composite film (PFHP) was fabricated, comprising a polypropylene (PP) outer layer, an irradiation-cured in situ acrylate (HD) intermediate layer, and a polyvinylidene fluoride (PVDF) outer layer. The HD layer, with its moderate dielectric constant (ε_r_ ≈ 4.2), mitigates the dielectric discontinuity and local electric field distortion at the PP/PVDF interface. Furthermore, in situ curing forms a dense interface and deep traps, thereby suppressing carrier migration and conduction losses. This structure synergistically combines the high polarization capacity of PVDF with the high breakdown strength and low loss characteristics of PP. Under a maximum test electric field of 450 MV m^−1^, the PFHP film achieved a discharge energy storage density of 4.09 J cm^−3^, with a charge–discharge efficiency of over 90%. This energy storage density was 2.13 times that of the pure PP film under the same electric field. Overall, the introduction of an acrylate interlayer not only mitigates the dielectric constant mismatch at the interface but also regulates charge transport by forming a cross-linked network, thereby suppressing leakage current and space charge accumulation, which helps improve energy storage performance. This work provides an effective strategy for developing all-organic thin-film capacitors with high energy storage capacity and high efficiency.

## 1. Introduction

Organic film capacitors offer advantages such as high voltage-withstanding capability, rapid charge and discharge response, and good operational stability, rendering them key components in pulse power supply systems, hybrid vehicles, and smart grids [1,2,3,4,5]. However, their relatively low energy-storage density severely limits their broader application in advanced power electronic systems. Additionally, a relatively large volume is often required to achieve the desired energy-storage capacity. For example, in hybrid vehicles, conventional biaxially oriented polypropylene (PP) film capacitors typically account for ~35% of the total inverter volume [6]. Since the energy storage performance of organic film capacitors depends primarily on their dielectric film, improving the dielectric constant and breakdown strength of the dielectric film is essential for increasing the energy-storage density.

Focusing on these two main areas, a variety of strategies have been explored to enhance the energy-storage density of dielectric membranes, including organic–inorganic composites, all-organic composites, and surface engineering [7,8,9,10,11,12,13]. For example, Xie et al. designed a hybrid filler structure in which hexagonal boron nitride (h-BN), which has a low dielectric constant and high breakdown strength, and barium titanate (BaTiO_3_), with a high dielectric constant and low breakdown strength, were uniformly dispersed within a PP matrix. Consequently, the dielectric constant increased from 2.2 to 2.92, whereas the breakdown strength (Eb) remained at 469 MV m^−1^, ultimately increasing the energy-storage density to 2.82 J cm^−3^ [14]. Additionally, Zhou et al. proposed a domain-type polarization mechanism that, by systematically regulating the core–shell structure, overcame the bottleneck whereby high dielectric constants are often accompanied by high losses, thereby achieving a breakthrough in comprehensive performance [15]. However, organic–inorganic composite strategies still suffer from certain inherent drawbacks, such as poor compatibility between the organic matrix and the inorganic fillers, and a tendency for filler agglomeration, which can significantly reduce the breakdown strength of the composite film. The addition of inorganic fillers can also lead to limited film processability, which is not conducive to large-scale production. In contrast, the all-organic composite strategy effectively resolves the mismatch between the dielectric and mechanical properties of organic–inorganic composites and is therefore attracting increasing research attention. For instance, Pan et al. constructed three-dimensional networks with varying degrees of stiffness by introducing three different organic small-molecule crosslinking agents that reacted with anhydride groups. Through deep-trap-level tuning and carrier trapping, the resulting material achieved an energy-storage density of 7.02 J cm^−3^ at an elevated temperature of 150 °C [16]. In another study, Feng et al. prepared an acrylonitrile–butadiene rubber/P(VDF-HFP) all-organic composite using a simple solution casting method followed by heat treatment. By controlling the smoothness of the film surface to reduce local electric field distortions, this composite material achieved an energy-storage density of 11.3 J cm^−3^ under an electric field of 510 MV m^−1^. Furthermore, multilayer structural design offers a novel approach to synergistically enhancing the performance of dielectric films. Specifically, this strategy enables the decoupled control of the dielectric constant and breakdown strength, thereby simultaneously improving both parameters and ultimately achieving a higher energy-storage density [17]. For example, He et al. placed highly polarized carbon nanotube fillers and highly insulating boron nitride nanosheet fillers in separate layers, thereby avoiding mutual interference between the two fillers within a single system, optimizing the composition of the multilayer structure, and achieving an energy-storage density of 3.57 J cm^−3^ (η ≈ 70%) [18]. Zhang et al. constructed an all-organic multilayer film by laminating high-dielectric-constant polyvinylidene fluoride (PVDF) with biaxially oriented polypropylene (BOPP), thereby doubling the discharge energy storage density (0.99 J/cm^3^) compared to pure BOPP under low-electric-field (200 kV/mm) and high-temperature (125 °C) conditions [19]. However, relying solely on a laminated structure of PP and PVDF to introduce high-dielectric polymers—while capable of increasing the dielectric constant—is typically accompanied by issues such as a decrease in breakdown strength, increased dielectric loss, and greater process complexity. Most of the layered composite films reported to date still fall short of ideal performance in terms of breakdown strength and charge/discharge efficiency. This is primarily due to sudden changes in the dielectric constant and space charge accumulation at heterogeneous interfaces, which lead to localized electric field concentration and increased energy loss, thereby limiting further improvements in energy storage density. It is therefore essential to develop layered composite films that combine high dielectric constants with high breakdown strengths.

This study therefore aims to develop a gradient dielectric constant composite film based on polyvinylidene fluoride (PVDF), which possesses a high dielectric constant, acrylate (HD), with a high breakdown strength, and biaxially oriented PP as the main materials. An in situ irradiation curing method is employed to integrate three film layers into a single composite film with a gradient dielectric constant. The resulting laminated structure is expected to generate a gradient distribution of the electric field within the film, thereby effectively mitigating the localized electric field concentration. Simultaneously, the interlayer interface is intended to act as a trap for charge carriers, shortening the distance they travel and thereby suppressing the leakage current. The proposed gradient dielectric constant composite film is designed to combine high energy-storage density with high charge–discharge efficiency, providing an effective strategy for the design of high-energy-storage polymer dielectric films.

## 2. Materials and Methods

The PVDF film was fabricated in-house using a laboratory-scale biaxial stretching process. The 4.8-micrometer PP film was purchased from Anhui Tongfeng Electronics Co., Ltd. (Tongling City, Anhui Province, China). High-dielectric-constant acrylate (HD) is 1,9-nonanediol dimethacrylate (molecular formula: C_17_H_28_O_4_, purity > 90%, density 0.968 g/cm^3^, boiling point 372.0 ± 15.0 °C, refractive index 1.46, flash point 173.9 ± 18.8 °C), purchased from Shanghai Aladdin Biochemical Technology Co., Ltd. (Shanghai, China). In this study, the HD layer consisted of pure 1,9-nonanediol dimethacrylate without any additional components.

PVDF and PP films were cut into rectangular sheets measuring 20 cm × 15 cm, which were then surface-cleaned and blown dry with N_2_. The PVDF film was laid flat on a clean glass substrate and secured at the four corners. A measured quantity of the HD monomer was evenly applied to the surface of the PVDF film using a squeegee and spread at a constant speed to form a liquid monomer layer of uniform thickness. The flattened PP film was then laid over the coating and smoothed again using a squeegee to remove air bubbles and ensure tight bonding between the layers. The samples described above were transferred to an irradiation source for curing. The electron beam energy was fixed at 150 keV, and the total irradiation doses were set at 10, 20, 30, and 40 kGy, respectively, to prepare PFHP-1, PFHP-2, PFHP-3, and PFHP-4. All samples were cured in a single step at room temperature in an air atmosphere. The electron beam system automatically controlled the exposure time based on the preset total dose, fixed beam current, and scanning parameters; therefore, dose rate and exposure time were not recorded as independent variables. Except for the total dose, all other irradiation parameters were kept consistent across all samples. Additionally, under the same conditions, a measured amount of HD monomer was cured into a film on an ITO glass substrate in a N_2_ atmosphere (to prevent oxygen-induced depolymerization). Sample thicknesses were measured using a proprietary thickness gauge; the PVDF film was approximately 12 μm thick, the PP film was 4.8 μm thick, and the PHFP sample was approximately 22 μm thick. Using vacuum deposition technology, square aluminum electrodes with a side length of 1.2 cm and an area of 1.44 cm^2^ were deposited on both sides of each film.

The surface and cross-sectional topographies of the composite films were observed using scanning electron microscopy (SEM; Zeiss Gemini SEM 500 SEM, GER). Attenuated total reflectance–Fourier transform infrared (ATR-FTIR) spectroscopy was performed using a Nicolet iZ10 FTIR spectrometer (Thermo Scientific, US) equipped with an ATR accessory to determine the functional groups present in the samples. Differential scanning calorimetry (DSC) curves were recorded using a DSC-Q2000 DSC (TA Instruments, US) from room temperature to 200 °C under a nitrogen atmosphere at a heating rate of 10 °C/min. A 4294A impedance analyzer (Agilent, US) was used to measure the dielectric constant and dielectric loss at room temperature in the frequency range of 100 Hz to 5 MHz. Additionally, using a Premier II-10 kV-1 multiferroic ferroelectric testing system (Radiant Technologies, US), the electric displacement–electric field (D–E) loop was recorded at a frequency of 10 Hz, and the leakage current density was recorded simultaneously. Thermally stimulated depolarization current (TSDC) measurements were performed using a Huace test system. The sample films were polarized for 20 min at voltages of 192 V and 880 V and a temperature of 60 °C (Samples 1 and 2 required a common polarization field), and then cooled to −50 °C at a rate of 15 °C/min. The temperature range for measuring the thermally excited depolarization current was −50 to 130 °C, with a heating rate of 5 °C/min. Prior to measurement, the sample film was short-circuited for 3 min to eliminate the effects of residual charge. To clarify the effect of the graded dielectric structure on the internal electric field distribution, a finite element simulation was performed using COMSOL Multiphysics 6.3 (AC/DC Module) under electrostatic conditions. The model was constructed as a three-layer stacked structure consisting of PP, HD, and PVDF, with thicknesses of 4.8, 5, and 12 μm, respectively. Based on low-frequency dielectric test results, the relative permittivities of PP, HD, and PVDF were set to 2.2, 4.2, and 11, respectively. A voltage corresponding to a nominal electric field of 200 MV m^−1^ was applied to the upper surface, while the lower surface was grounded. A free triangular mesh was used, with local refinement near the interfaces. Mesh convergence tests showed that the maximum change in the electric field was less than 1% after further mesh refinement. All layers were treated as ideal insulators. To evaluate the energy storage and discharge characteristics of the prepared films, the discharge behavior was characterized using a PKDIS10012 capacitor discharge test system (PolyK Technologies, US) under an electric field of 200 MV m^−1^ and a load resistance of 100 kΩ. All dielectric, leakage current, and P–E measurements were performed at room temperature (approximately 28 °C), with relative humidity maintained at around 60%.

## 3. Results and Discussion

**Figure 1:**
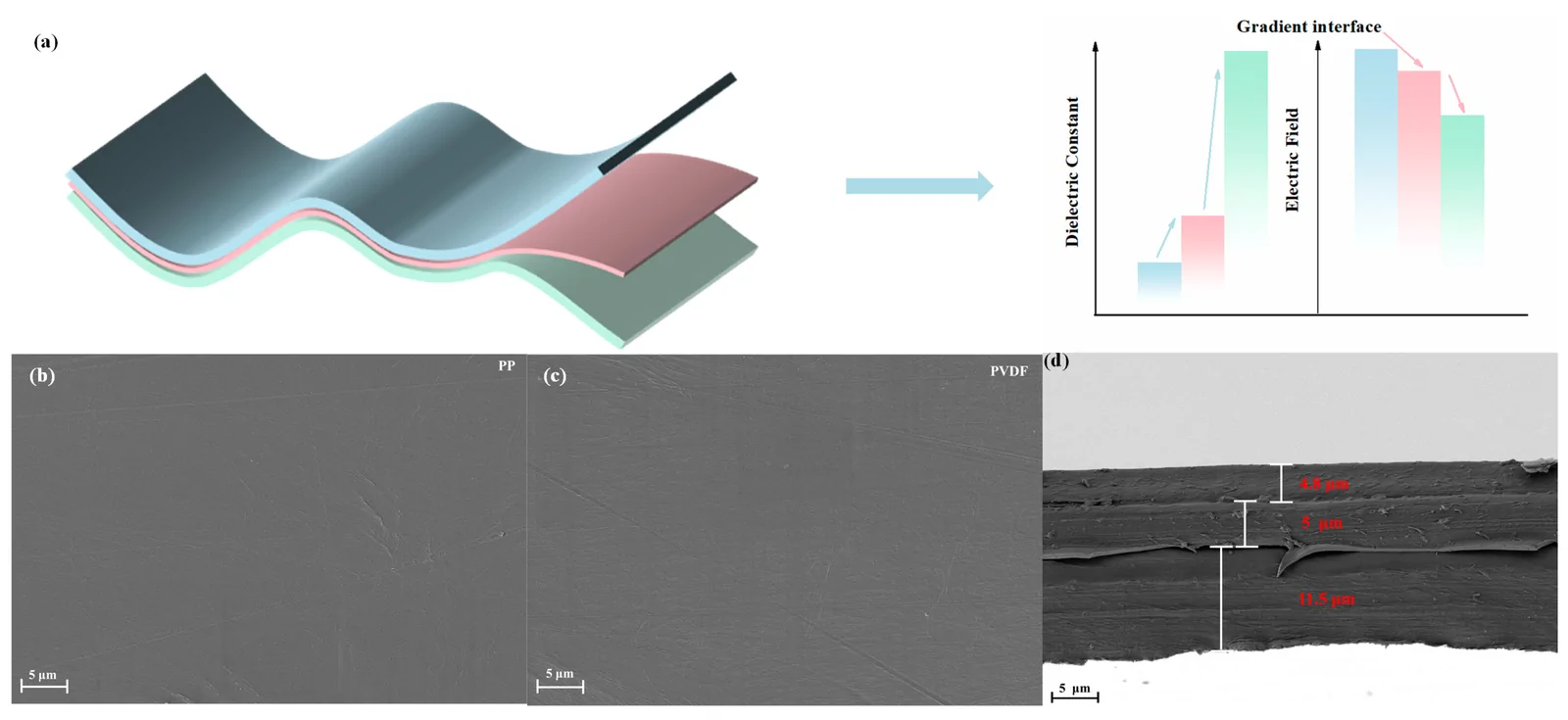
(**a**) Schematic illustration showing the design of the dielectric constant gradient in a sandwich-structured film and its modulation of the local electric field. SEM images of the surface micro-topography of (**b**) the PP side and (**c**) the PVDF side. (**d**) Cross-sectional morphology of the film.

A schematic illustration of the design of the gradient sandwich-structured film is shown in Figure 1a, where the outer layer consists of a PP film, the middle layer consists of an HD film, and the bottom layer consists of a PVDF film. Unlike the two- and three-layer PP/PVDF structures reported in the literature, a sharp change in the electric field occurs at the interface where PP and PVDF are in direct contact, owing to the significant difference in the dielectric constants of the two phases [20]. In the PFHP film designed in this work, an HD layer was introduced as an intermediate buffer layer. Through a gradient design, the difference in dielectric constants was reduced layer by layer, and the overall breakdown voltage was increased by homogenizing the electric field. Furthermore, the multiple interfaces in the sandwich structure slow down carrier migration and suppress charge injection, thereby enhancing the film’s breakdown resistance. As shown in Figure 1b–d, the surfaces of both the PP and PVDF films are relatively flat and smooth, with no obvious defects such as particles or voids observed. Cross-sectional morphology reveals that the HD layer is uniformly and tightly bonded between the PP and PVDF layers; there are no obvious gaps at the interface, and the interface is morphologically continuous.

**Figure 2:**
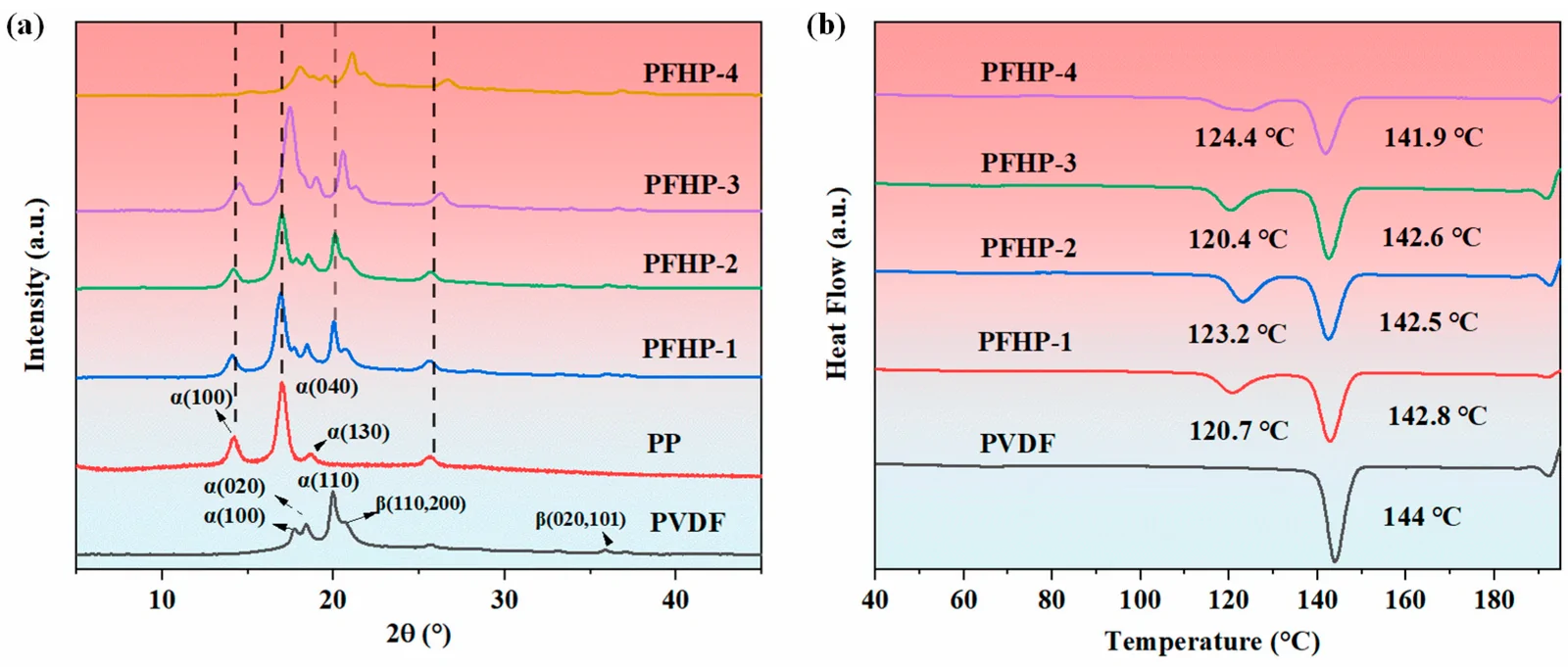
(**a**) XRD patterns and (**b**) DSC curves of the sandwich-structured films and their constituent materials.

The film samples were characterized using ATR-FTIR, and the results are shown in Figure S1. Owing to the limitations imposed by the sample thickness and ATR detection depth, only IR signals corresponding to the surface PP layer were detected in the PFHP samples. Figure S2 shows the Fourier transform infrared spectra of HD films prepared under identical conditions. The results indicate that, following electron beam irradiation, both the C = C stretching absorption peak at 1635 cm^−1^ and the out-of-plane bending vibration peak near 810 cm^−1^ (typically attributed to =C–H) were attenuated. This suggests that the acrylic ester double bonds have been consumed, thereby confirming that polymerization/cross-linking of HD has occurred. Additionally, the crystal structures and crystallization behaviors of the composite films with gradient sandwich structures were analyzed using X-ray diffractometry (XRD) and DSC. Figure 2a shows the XRD patterns of the various samples, in which the diffraction peaks at 17.6°, 18.3°, 19.9°, and 20.3° correspond to the α(100), α(020), α(110), and β(110,200) crystal planes of PVDF, whereas the peaks at 14.0°, 16.8°, and 18.5° correspond to the α(110), α(040), and α(130) crystal planes of PP [21]. The XRD pattern of the PFHP film showed characteristic diffraction peaks originating from both PP and PVDF, indicating that the crystalline structures of both components were preserved in the composite film. No other peaks were observed, likely due to the amorphous structure of the HD layer. The DSC cooling curves recorded for the prepared samples are shown in Figure 2b. The pristine PVDF film exhibited a major exothermic crystallization peak at ~144 °C, while the PFHP film exhibited a new low-temperature crystallization peak between 120 and 124 °C. This peak was considered to originate either from the crystallization behavior of the HD/PP layer or from an interface-induced crystallization effect. Notably, the temperature of the main crystallization peak of PVDF in the gradient sandwich-structured film decreased slightly, suggesting that the HD/PP layer influenced the crystallization process of PVDF.

**Figure 3:**
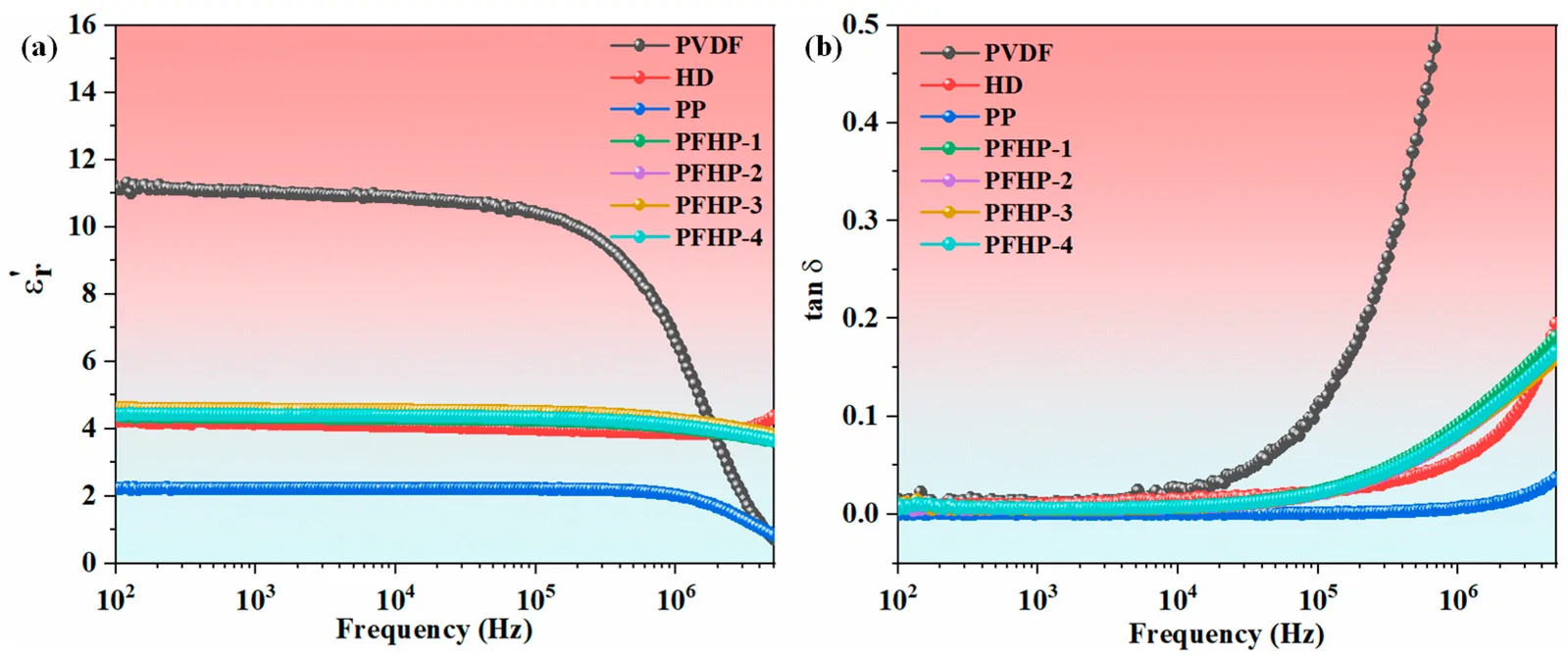
Frequency-dependent spectra of (**a**) the dielectric constant and (**b**) the dielectric loss of the gradient sandwich-structured films and their constituent materials.

To further investigate the influence of the gradient structure on the dielectric properties of the composite films, the frequency dependence of the dielectric constant and dielectric loss of each film was measured using broadband dielectric spectroscopy, and the results are presented in Figure 3a,b. At a frequency of 1 kHz, the PVDF monolayer film exhibited the highest dielectric constant (~11), the PP monolayer film exhibited the lowest dielectric constant (~2.2), and the HD monolayer film exhibited an intermediate value (~4.2). According to the series capacitance model, the effective dielectric constant of the gradient sandwich-structured film is expected to lie between those of the individual components. Additionally, the measured dielectric constant of the PFHP film was ~4.5, representing a significant improvement over that of the pure PP film. Figure 3b shows the frequency dependence of the dielectric loss. Although PVDF films possess high dielectric constants, their loss at 1 kHz is relatively high (~0.015), which may limit their practical applications. In contrast, PP films exhibit an extremely low loss (~0.0004), whereas HD films exhibit a relatively low loss (~0.003). As a result, the PFHP gradient-structured film exhibits a loss of 0.005 at 1 kHz, indicating that the composite film has achieved a balance between dielectric constant and dielectric loss. Furthermore, the gradient sandwich structure effectively minimized the sharp increase in the loss of the PVDF film at high frequencies, thereby improving the operational stability of the device under high-frequency conditions. Specifically, at 1000 Hz, the loss of PVDF was 0.010, while that of PFHP-3 was 0.005. When the frequency increased to 104, the loss of PVDF was 0.026, and that of PFHP-3 was 0.006. Finally, when the frequency rose to 106, the loss of PVDF was 0.686, while that of PFHP-3 is 0.079. These data indicate that the dielectric loss of PVDF increases quite sharply, whereas the increase for PFHP-3 is relatively small. This quantitatively demonstrates that the PFHP structure can effectively suppress the rise in dielectric loss of PVDF at high frequencies.

**Figure 4:**
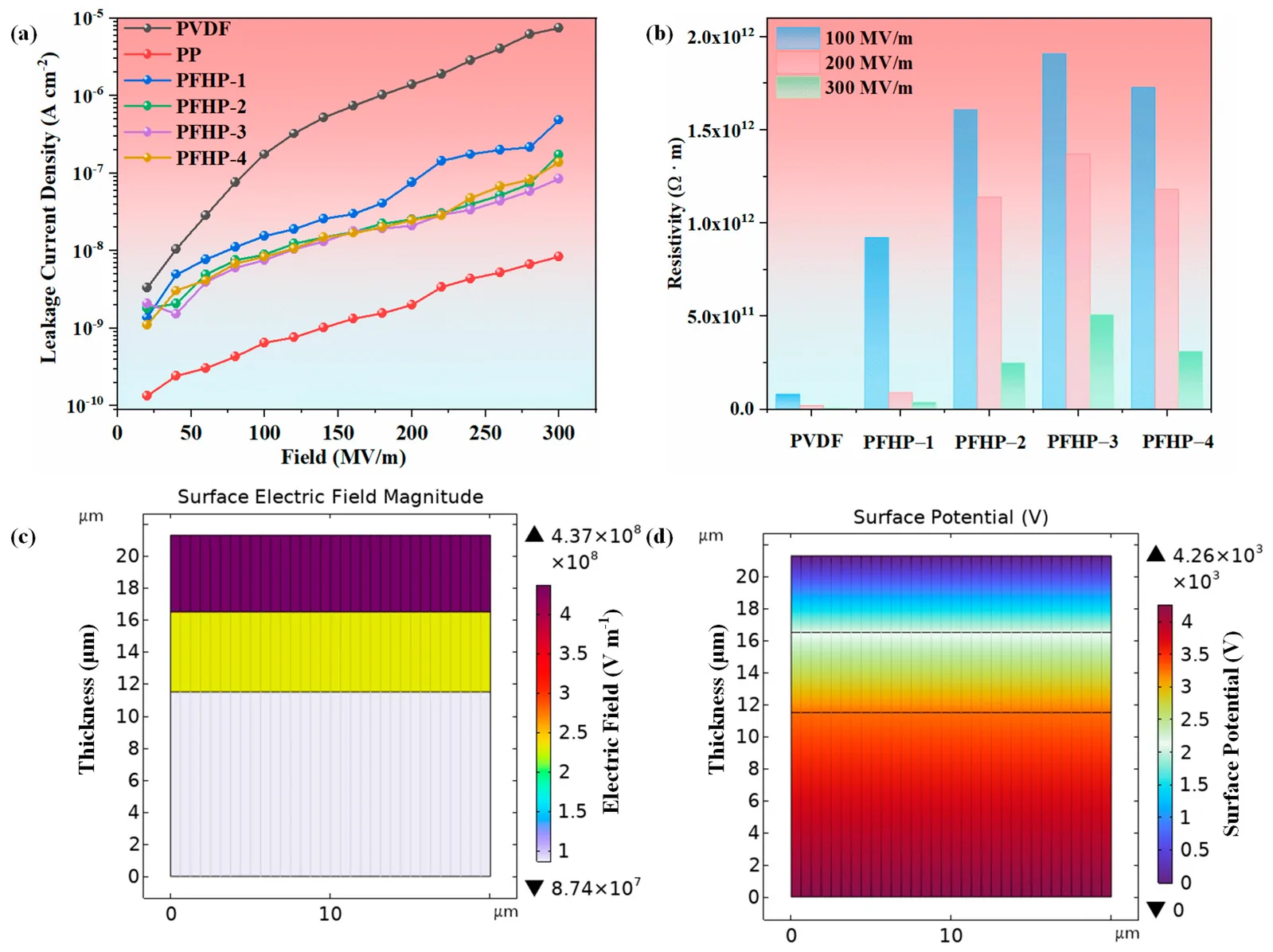
(**a**) Leakage current and (**b**) insulation resistivities of PP, PVDF and PFHP films. (**c**) Electric field and (**d**) electric potential for the PFHP film.

Subsequently, the leakage currents of the films were measured to evaluate their electrical insulation properties. As shown in Figure 4a, the PVDF film exhibited a high leakage current, the PP film exhibited a low leakage current, and the leakage currents of the gradient sandwich-structured films were significantly lower than that of the PVDF film. These results indicate that the interfaces within the gradient multilayer structure effectively slowed carrier migration, thereby suppressing the leakage current. Additionally, Figure 4b shows the insulation resistivities of the films under varying electric fields. Since the resistivity of the PP film is significantly higher than that of the other samples, this figure compares only the insulation resistivities of the PVDF and PFHP samples. As shown, the PFHP films tend to exhibit higher insulating resistivity than the PVDF film, with the PFHP-3 sample providing the highest value. Furthermore, Figure 4c shows a simulation of the local electric field distribution in a gradient sandwich-structured film under an applied electric field of 200 MV m^−1^. Due to differences in the dielectric constants of the three layers (PVDF ~11, HD ~4.2, PP ~2.2), and based on the equivalent series model, the electric field exhibits a significant non-uniform distribution along the thickness direction. Specifically, the PVDF layer exhibits the lowest electric field (~87.7 MV m^−1^), the HD layer exhibits an intermediate electric field (~229.6 MV m^−1^), and the PP layer exhibits the highest electric field (~438 MV m^−1^). These results indicate that the gradient sandwich structure successfully facilitated the effective transfer of the electric field from the high-dielectric-constant layer to the low-dielectric-constant layer, thereby concentrating the high electric field within the PP layer, which possesses a higher intrinsic breakdown field strength. This redistribution of the electric field reduces the risk of electrical fatigue in the PVDF layer and enhances the breakdown resistance of the composite film. Moreover, Figure 4d shows the simulation results for the corresponding potential distribution. The potential decreases monotonically with thickness and is continuous without any jumps at the interlayer interfaces.

**Figure 5:**
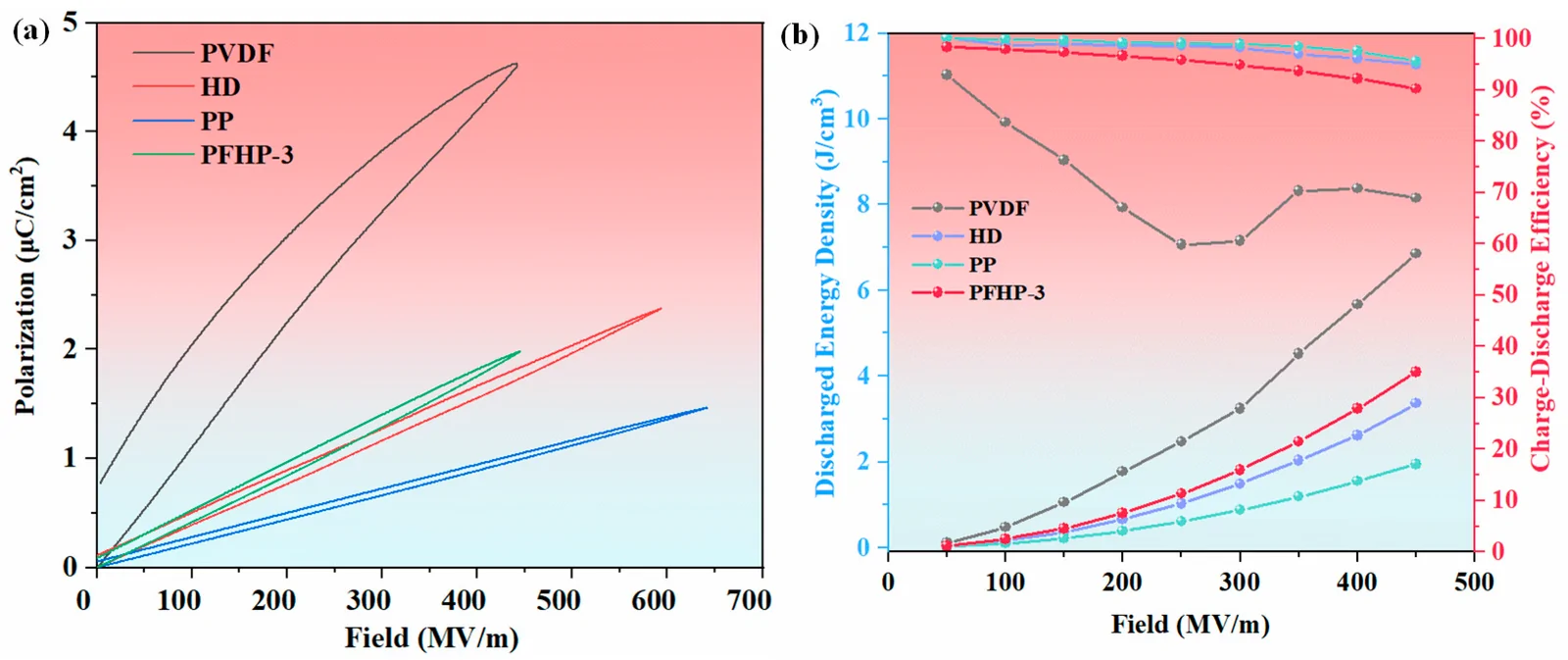
(**a**) Comparison of the maximum polarization values, and (**b**) comparison of the energy-storage density and charge–discharge efficiency for the PFHP-3 film and the corresponding monolayer films.

Based on the polarization–electric field (P–E) curve at the maximum electric field (Figure 5a, see Figure S3 for the complete loop), the energy-storage density and charge–discharge efficiency were calculated for the PFHP-3 gradient sandwich-structured film and the respective monolayer films under high electric fields (according to Equations (S1)–(S4)). The PVDF film exhibits typical relaxor ferroelectric behavior, with a maximum polarization of 4.6 μC cm^−2^, which is significantly higher than the corresponding values of the other films. Due to limitations in film thickness, PFHP films can achieve a maximum electric field of ~450 MV m^−1^ at a test voltage of 10,000 V, corresponding to a maximum polarization of 1.96 μC/cm^2^. Notably, this value is significantly higher than the maximum polarization value of the PP film, providing a foundation for PFHP films to achieve higher energy-storage densities. More importantly, according to the equivalent series model, the PP layer is subjected to a higher electric field, whereas the P–E curve of the PFHP-3 film remains approximately linear, indicating that the PFHP-3 film exhibits a relatively higher charge–discharge efficiency under the maximum test electric field.

The charge–discharge efficiency is a key parameter for evaluating film capacitors and is directly related to the energy loss and heat generation of the device. Figure 5b compares the energy storage densities and charge–discharge efficiencies of the various films at the maximum test electric field for the PFHP-3 film (450 MV m^−1^). Although the PVDF film exhibited the highest energy-storage density, its charge–discharge efficiency initially decreased and then slowly recovered with increasing electric field, reaching 68% at the maximum electric field. In comparison, the PP and HD films exhibited lower energy storage densities but higher charge–discharge efficiencies. Thus, owing to its three-layer structure, the PFHP-3 film leverages the synergistic advantages of its constituent materials. Under the maximum measurable electric field, its energy-storage density reaches 4.09 J cm^−3^, which is 2.13 times higher than that of the PP film under the same electric field. More importantly, while achieving a high energy-storage density, the charge–discharge efficiency of the PFHP-3 film remained above 90%, indicating that the increase in energy-storage density was achieved without sacrificing the charge–discharge efficiency. At the same time, to further verify the reliability of the maximum energy storage density, we conducted multiple repeat tests on independently prepared samples. The results showed that the high energy storage density was consistently reproducible across different batches of samples, and the variation in test results was minimal. This indicates that this performance is not a random outcome of a single test, but rather a reflection of the samples’ intrinsic properties (Table S2). The gradient sandwich-structured film utilizes the high polarization capability of PVDF arising from its high dielectric constant and combines it with the high charge–discharge efficiency characteristics of the linear dielectric PP and HD films. This strategy of constructing films through a gradient dielectric constant design provides a viable approach for developing all-organic dielectric films that combine high energy-storage density with high charge–discharge efficiency.

**Figure 6:**
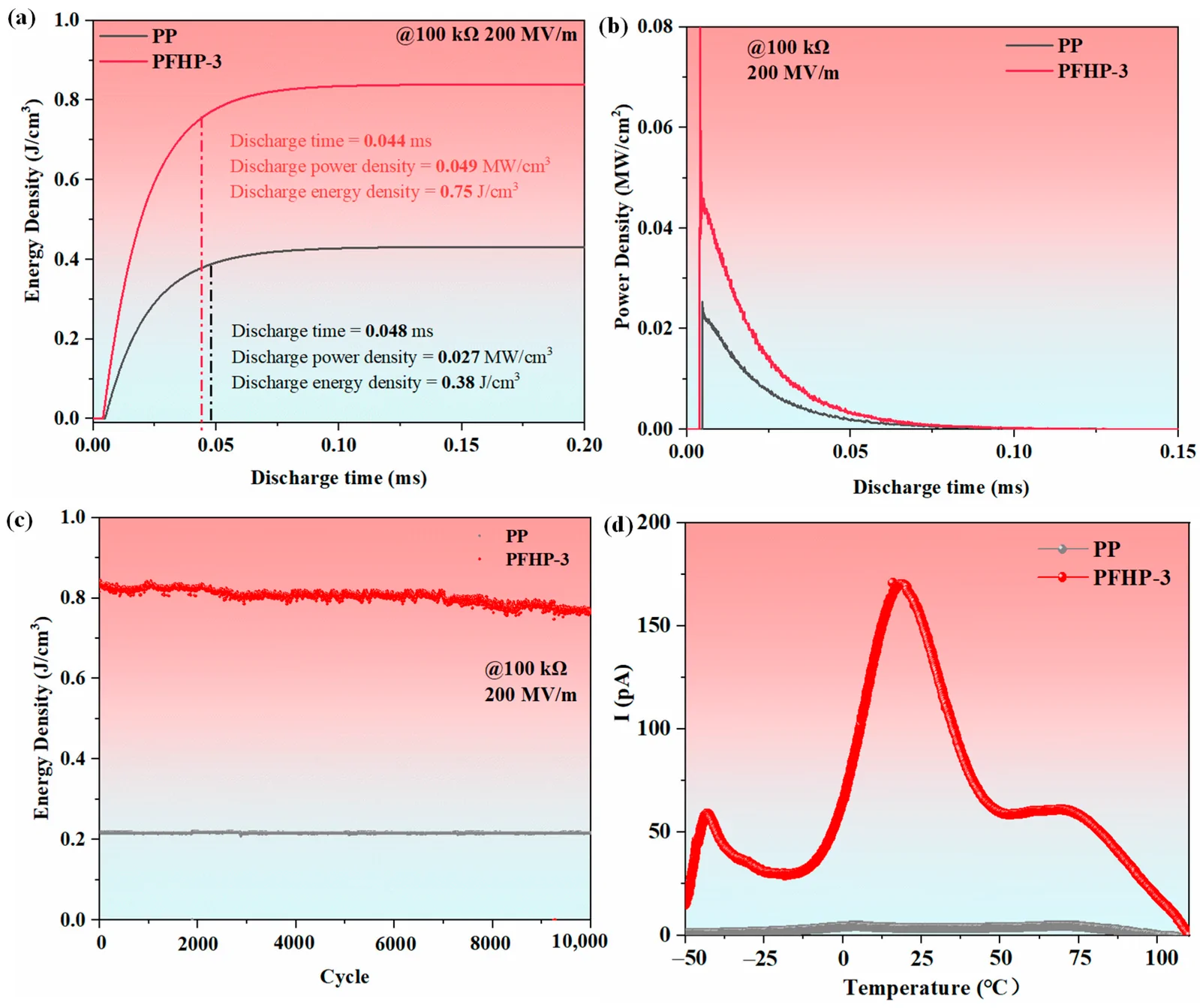
(**a**) Charge–discharge energy density and (**b**) power density as a function of time for the PFHP-3 and PP films. (**c**) Cycling stability comparison over 10,000 cycles, and (**d**) TSDC spectra of the PFHP-3 and PP films.

In high-power pulsed-capacitor applications, rapid charging and discharging are of paramount importance [22,23]. Based on Table S1, we compared the relevant properties of PFHP films under an electric field of 400 MV m^−1^. Among them, PFHP-3 exhibited the highest average discharge energy density. Although the differences compared to the other samples were minor, it was still selected as the representative sample for further detailed characterization. Thus, using a capacitor discharge test system, the discharge rates and power densities of the PFHP-3 and PP films were compared under a load resistance of 100 kΩ. As shown in Figure 6a, under a charging electric field of 200 MV m^−1^, the PP film released an energy density of 0.38 J cm^−3^ within 0.048 ms, while the PFHP gradient sandwich-structured film released a higher energy density of 0.75 J cm^−3^ within 0.044 ms. Additionally, the maximum power density of the PFHP-3 film reached 0.049 MW cm^−3^ (Figure 6b), which is also significantly higher than that of the PP film (i.e., 0.027 MW cm^−3^). This indicates the potential of the PFHP-3 film for application in high-energy-density pulse capacitors.

Cycle stability is another key factor that determines the practical applications of films. Charge–discharge cycle tests were therefore performed on the PP and PFHP-3 films under an electric field of 200 MV m^−1^ and a load of 100 kΩ. As shown in Figure 6c, the discharge energy density of the PP film remained essentially stable after 10,000 charge–discharge cycles. In contrast, the discharge energy density of the PFHP film decreased slightly (~7.2%) after 10,000 cycles, likely due to ferroelectric hysteresis losses and space charge migration losses in PVDF. To investigate the underlying mechanism responsible for this observation, TSDC characterization was performed on the PP reference membrane and the PFHP-3 membrane, and the results are shown in Figure 6d. The trap energy depth (Et) and trap charge (Q) were calculated using Equations (S5) and (S6) [24]. TSDC results show that the PFHP-3 film exhibits two peaks near approximately 18 °C and 75.2 °C, corresponding to apparent trap energy levels (or relaxation activation energies) of 0.591 eV and 0.861 eV, respectively, and trap charges of 40.71 nC and 7.14 nC, respectively. Since the TSDC peaks of the composite film are broad and overlap to some extent, the above values should more accurately be regarded as apparent trap energy levels or relaxation activation energies, rather than being directly interpreted as single, discrete deep trap energy levels. The presence of these deep trap states suggests that the HD interlayer may have a passivating effect on the shallow-level traps at the PVDF/PP interface and introduced a certain number of deep-level traps, thereby helping to suppress carrier migration and delay the formation of breakdown channels. However, it should be noted that the existing data cannot yet directly prove that the deep traps originate entirely from the HD interlayer or the interface. The above explanation remains a reasonable conjecture and requires further confirmation through more systematic control experiments and more detailed peak separation analysis.

Table 1 summarizes a comparison of the key performance characteristics of PP-based dielectric films reported in recent years with those of this work. As shown, by constructing a PP/HD/PVDF sandwich structure with a gradient dielectric constant, this work achieved a discharge energy storage density of 4.09 J cm^−3^ without introducing any inorganic fillers. Due to limitations imposed by the maximum output voltage of the testing system, we were unable to evaluate the material’s ultimate energy storage performance under higher electric fields. However, the obtained performance still demonstrates a good overall balance, achieving a harmonious compromise among discharge energy density, high charge–discharge efficiency, low dielectric loss, and a moderate dielectric constant. Specifically, the gradient-structured film prepared in this study exhibits a dielectric constant of approximately 4.5, which is relatively high among similar PP-based films. This is primarily attributable to the introduction of the HD intermediate layer and the resulting gradient in dielectric constant along the thickness direction. At the same time, the 90% charge–discharge efficiency outperforms a significant number of comparison systems, indicating that this structure can effectively suppress polarization loss and space charge accumulation, thereby maintaining high energy release capacity under high electric fields. Therefore, although the energy storage density achieved in this work is not the highest among all comparison systems, its advantage lies in achieving a good balance between energy storage performance, dielectric constant, charge–discharge efficiency, and losses without the need to add high-dielectric inorganic fillers, demonstrating good potential for practical applications. This result also provides a foundation for further optimization of the gradient structure and for increasing the test electric field strength in future work.

## 4. Conclusions

In summary, using irradiation-induced in situ curing technology, a fully organic composite film with a PFHP sandwich structure and a gradient dielectric constant was prepared. Both experimental and simulation results indicate that the design of the gradient dielectric constant effectively mitigates the electric field concentration caused by sudden changes in the dielectric constant, while the high-quality interface formed by the in-situ curing of the HD layer introduces deep energy level traps, thereby enhancing the composite film’s carrier capture capability and suppressing carrier migration. Under a maximum test electric field of 450 MV m^−1^, the film achieved an energy storage density of 4.09 J cm^−3^, while the charge–discharge efficiency remained consistently above 90%. Admittedly, this work still has limitations, including constraints on the test electric field, the lack of high-temperature performance evaluation, a limited number of cycles, and the failure to achieve large-scale fabrication. In future work, we will fabricate thinner films by biaxially stretching thinner PVDF. We will further validate and optimize this system through high-temperature and long-term cycling characterization, exploration of roll-to-roll processes, and direct space charge measurements.

## Figures and Tables

**Table 1:** Comparison of the dielectric and energy storage properties of the polymer-based dielectric film developed in this study with those of polymer-based dielectric films reported in recent years.

Sample	Dielectric Constant	Breakdown Strength (MV m^−1^)	Energy Storage Density (J cm^−3^)	Charge -Discharge Efficiency (%)	References
PP/BT/BN	0.92	469	2.82	**	[14]
PVA/BT@BOPP	3.2	408	2.90	83	[25]
PMMA@BT	3.75	448	3.86	94.1	[26]
PP-g-MAH/MMT	3.55	530	5.21	94.9	[27]
PTFE/PP	2.2	462	4.5	**	[28]
BO(PP/AA/AONs)	2.43	864	9.64	90	[29]
PP/PMMA Double-Layer	2.5	**	5.93	87	[30]
BOPP/PVDF/BOPP	2.77	690	5.71	84	[31]
PFHP-3	4.5	**	4.09	90	This work

Note: ** indicates that this parameter was not reported in the original literature. All other values are taken from the corresponding literature.

## Data Availability

The original contributions presented in this study are included in the article/Supplementary Materials. Further inquiries can be directed to the corresponding authors.

## Supplementary Materials

The following supporting information can be downloaded at https://www.mdpi.com/article/10.3390/polym18182202/s1.
