# Supplementary material for: Electric Field Modulation Enables High Energy-Storage Density and Low Loss in All-Organic Polymer Films with Gradient Dielectric Constants
Source: Polymers (Basel). 2026 Sep 10;18(18):2202. doi: 10.3390/polym18182202 (PMC13611204; doi:10.3390/polym18182202)
Supplement: Supplementary file 1 [file polymers-18-02202-s001.zip › polymers-4536701-supplementary.pdf]

Article

Electric Field Modulation Enables High Energy-Storage Density and Low Loss in All-Organic Polymer Films with Gradient Dielectric Constants

Jianfeng Li [1], Zerui Li [1], Fujia Chen [2], Yujiu Zhou [2], Hu Ye [1,2], Zhenyi Qu [3], Yuetao Zhao[4,*] and Jianhua Xu [1,*]

[1] School of Optoelectronic Science and Engineering, University of Electronic Science and Technology of China, Chengdu 610054, China; ljf1510830@163.com (J.L.); zeruili022@163.com (Z.L.); chem24@163.com (H.Y.)
[2] Department of Film Capacitors, Xinyun Electronic Components Company of China ZhenHua Group, Guiyang 550018, China; edc0402@163.com (F.C.); 18782941993@163.com (Y.Z.)
[3] School of Creative Design, Chongqing City Vocational College, Chongqing 402160, China; qzyym1217@163.com
[4] Ocean College, Jiangsu University of Science and Technology, Zhenjiang 212100, China
* Correspondence: jianhuaxu215@163.com (J.X.); zhaoyuetao@just.edu.cn (Y.Z.)

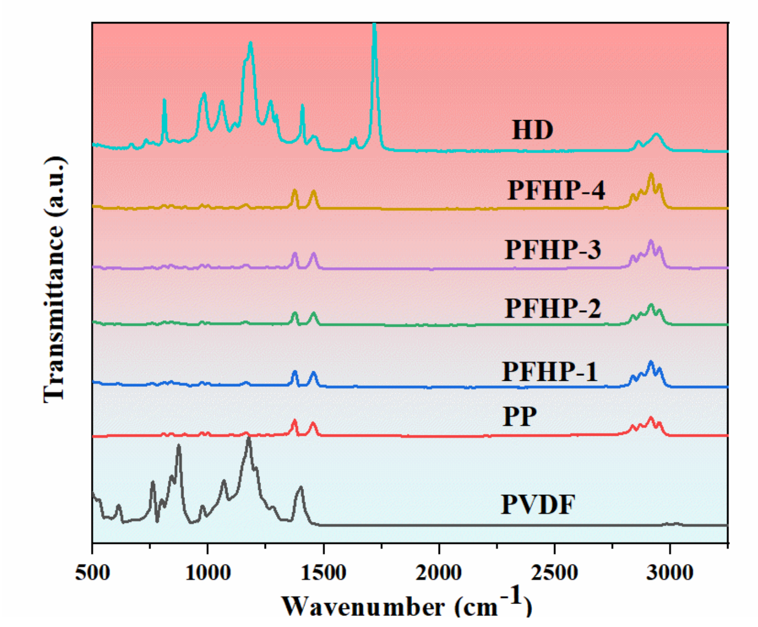

Figure S1. FT-IR spectra of the samples

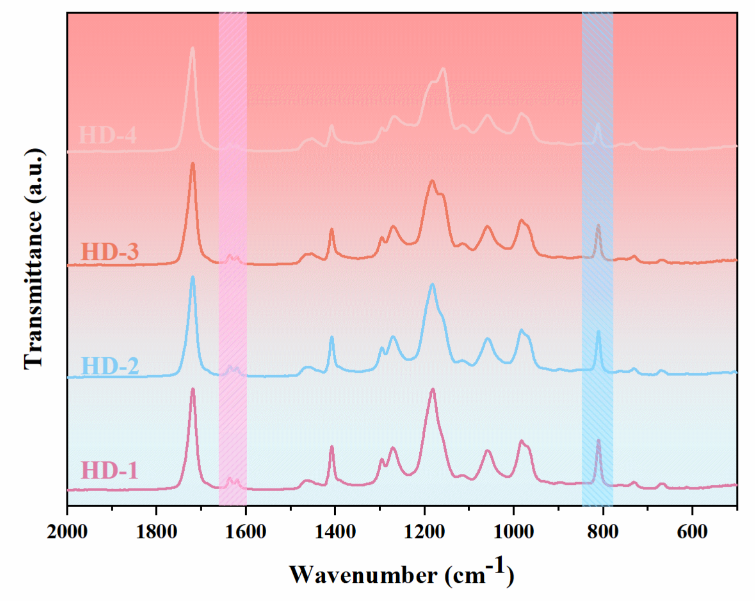

Figure S2: FTIR spectra of HD monomers coated on an ITO glass substrate before and after electron beam irradiation.

Calculate the energy storage density of the P-E line using the following formula:

$$U_{charge} = \int_{0}^{P_{max}} EdP \quad \text{S1}$$

$$U_{discharge} = \int_{P_{max}}^{P_{rem}} EdP \quad \text{S2}$$

$$U = \frac{1}{2}\varepsilon_0\varepsilon_r E^2 \quad \text{S3}$$

$$\eta = \frac{U_{discharge}}{U_{charge}} \times 100\% \quad \text{S4}$$

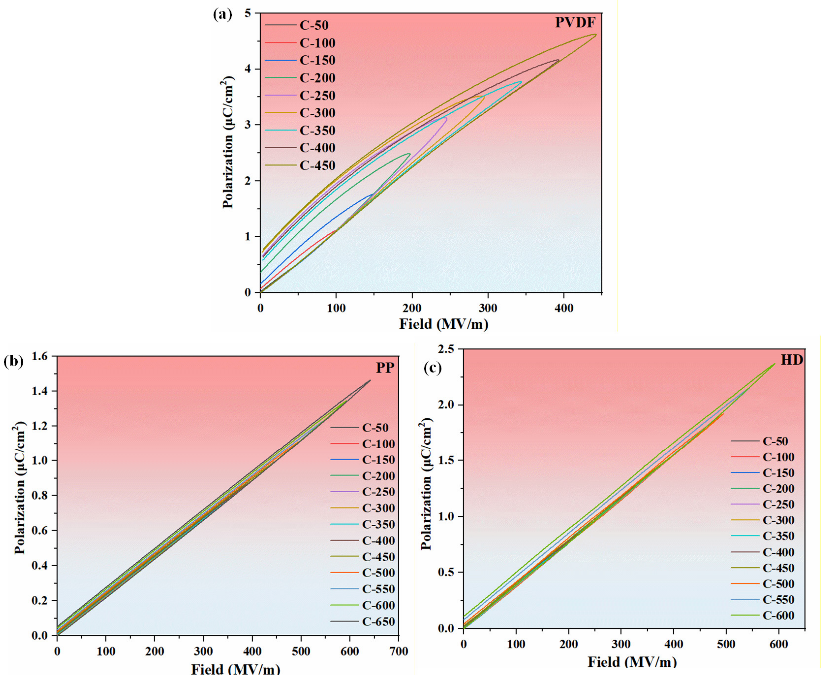

Figure S3. P-E characteristic curves for PVDF, PP and HD

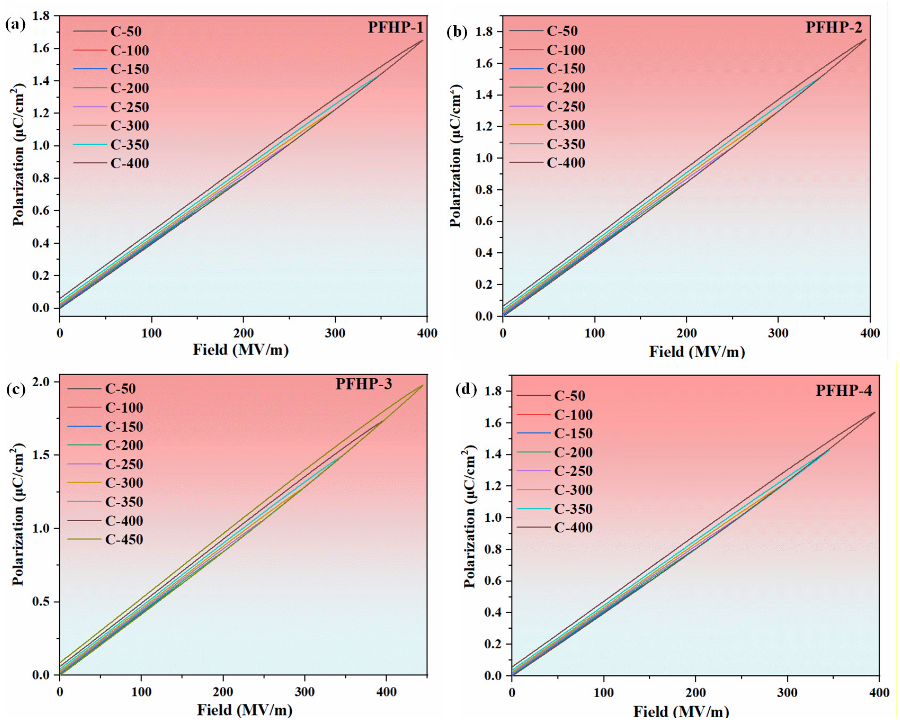

Figure S4. P-E loops curves for PFHP samples

Table S1. Maximum polarization, discharged energy density, and charge–discharge efficiency of PFHP-1–PFHP-4 at an electric field of 400 MV m^{-1}.

**Sample Name**	**Maximum polarization value (Pmax) (μC/cm²)**	**Energy Storage Density (Ue) (J/cm³)**	**Charge -Discharge Efficiency(η) (%)**
**PFHP-1**	1.65	3.07	91.65
**PFHP-2**	1.70	3.19	91.77
**PFHP-3**	1.73	3.24	92.19
**PFHP-4**	1.67	3.12	91.91

Table S2. Maximum polarization intensity, discharge energy density, and charge-discharge efficiency of PFHP-3 under the maximum applied electric field (repeatability test)

**Sample Name**	**Maximum polarization value (Pmax) (μC/cm²)**	**Energy Storage Density (Ue) (J/cm³)**	**Charge -Discharge Efficiency(η) (%)**
**1**	1.85	3.89	89.96
**2**	1.93	4.02	89.89

**3**	1.98	4.10	89.72
**4**	1.87	3.92	90.64
**5**	1.82	3.78	89.75
**6**	1.79	3.76	89.73
**7**	1.83	3.88	90.29
**8**	1.85	3.87	89.98
**9**	1.94	4.04	90.01
**10**	1.85	3.90	90.84

$$E_t = \frac{2.47 k_B T_p^2}{\Delta T} \quad \text{S5}$$

$$Q_t = \frac{1}{\beta}\int_{T_1}^{T_2} I(T)dT \quad \text{S6}$$
